# Supplementary material for: An antisense RNA capable of modulating the expression of the tumor suppressor microRNA-34a
Source: Cell Death Dis. 2018 Jul 3;9(7):736. doi: 10.1038/s41419-018-0777-5 (PMC6030072; doi:10.1038/s41419-018-0777-5)
Supplement: Supplementary file 1 — Supplementary Document 1 [file 41419_2018_777_MOESM1_ESM.pdf]

A)

cancer	all n	all rho	all p	TP53wt n	TP53wt rho	TP53wt p	TP53mut n	TP53mut rho	TP53mut p
Adrenocortical carcinoma (ACC)	10	0.55	1.04e−01	10	0.55	1.04e−01	NA	NA	NA
Bladder Urothelial Carcinoma (BLCA)	228	0.51	7.89e−17	134	0.45	3.86e−08	94	0.43	1.73e−05
Breast invasive carcinoma (BRCA) Basal	42	0.57	9.54e−05	10	0.62	6.02e−02	32	0.57	7.41e−04
Breast invasive carcinoma (BRCA) Her2	44	0.15	3.39e−01	12	0.22	4.85e−01	32	0.07	7.10e−01
Breast invasive carcinoma (BRCA) LumA	199	0.34	8.22e−07	177	0.34	2.96e−06	22	0.49	2.31e−02
Breast invasive carcinoma (BRCA) LumB	70	0.17	1.57e−01	61	0.15	2.53e−01	9	0.17	6.78e−01
Cervical squamous cell carcinoma and endocervical adenocarcinoma (CESC)	156	0.14	8.37e−02	145	0.16	5.45e−02	11	−0.05	9.03e−01
Head and Neck squamous cell carcinoma (HNSC)	313	0.54	8.38e−25	123	0.61	0.00e+00	190	0.45	9.68e−11
Kidney Chromophobe (KICH)	5	0.60	3.50e−01	5	0.60	3.50e−01	NA	NA	NA
Kidney renal clear cell carcinoma (KIRC)	142	0.35	2.06e−05	141	0.34	4.41e−05	NA	NA	NA
Kidney renal papillary cell carcinoma (KIRP)	167	0.45	9.16e−10	163	0.45	2.04e−09	4	0.80	3.33e−01
Brain Lower Grade Glioma (LGG)	271	0.63	9.92e−32	76	0.73	0.00e+00	195	0.39	2.26e−08
Liver hepatocellular carcinoma (LIHC)	153	0.56	3.64e−14	114	0.52	4.18e−09	39	0.45	3.95e−03
Lung adenocarcinoma (LUAD)	234	0.28	1.15e−05	128	0.36	2.87e−05	106	0.23	1.91e−02
Lung squamous cell carcinoma (LUSC)	139	0.23	6.74e−03	42	0.04	7.93e−01	97	0.33	9.91e−04
Ovarian serous cystadenocarcinoma (OV)	56	0.23	8.37e−02	10	0.84	4.46e−03	46	0.15	3.31e−01
Prostate adenocarcinoma (PRAD)	413	0.47	1.33e−23	375	0.46	6.13e−21	38	0.45	4.58e−03
Skin Cutaneous Melanoma (SKCM)	165	0.65	5.43e−21	152	0.61	7.85e−17	13	0.43	1.40e−01
Stomach adenocarcinoma (STAD)	225	0.37	8.23e−09	145	0.37	5.71e−06	80	0.42	1.03e−04
Thyroid carcinoma (THCA)	469	0.46	1.07e−25	467	0.46	4.06e−26	NA	NA	NA

B)

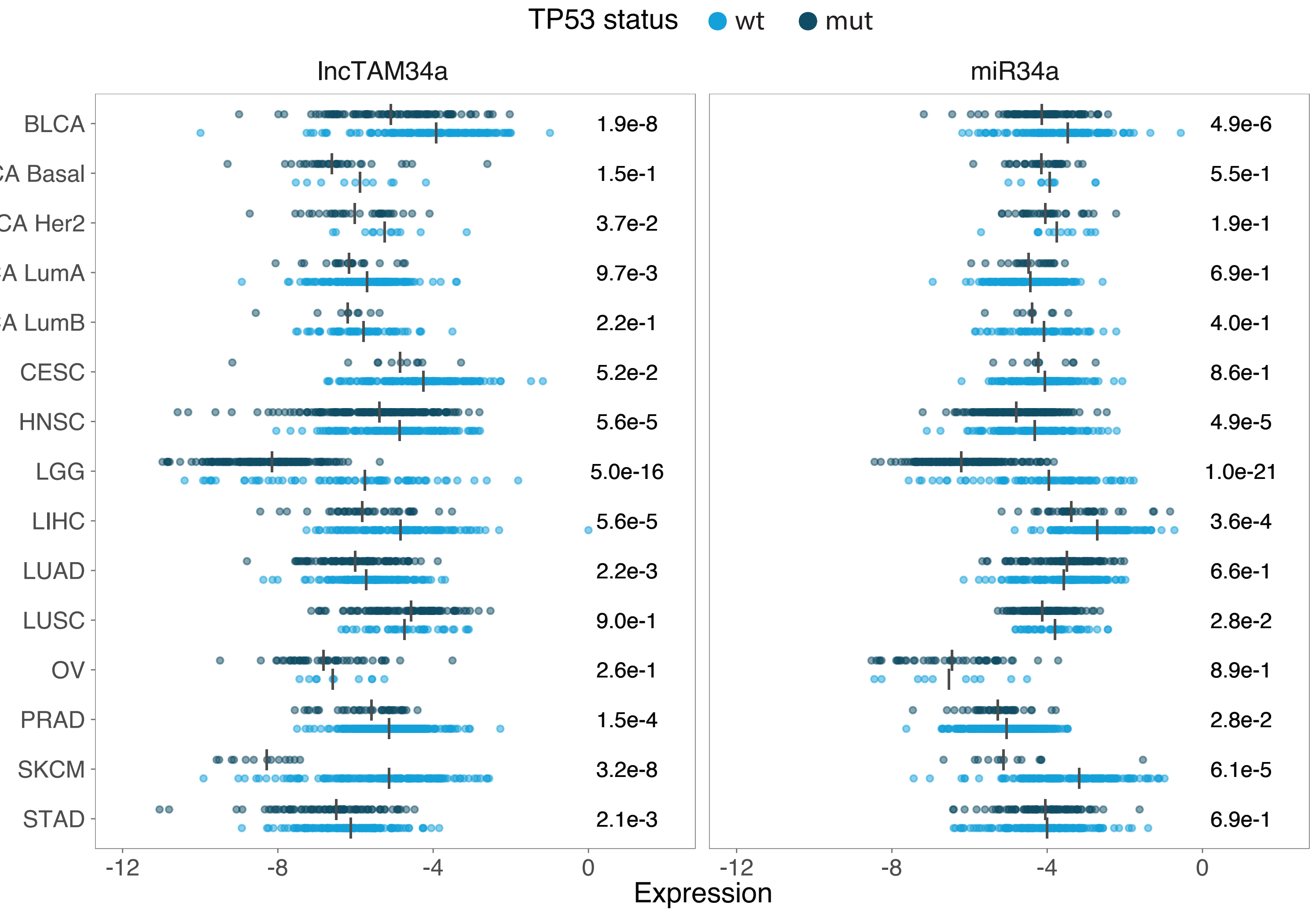

Supplementary Figure 1: A) Spearman's rho and P values (p) from the correlation analysis in Figure 1a between miR34a and lncTAM34a expression in TP53 wild type (wt) and mutated (mut) samples within TCGA cancer types. NA indicates not applicable, due to a lack of data for the specific group. **B)** Expression levels of miR34a and lncTAM34a in TP53 wt and nonsynonymous mutation samples. Expression was quantified by the log2 ratio of expression of the gene to its maximal expression value. Vertical lines indicate the median. P values are indicated on the right side of each panel and are derived from comparing the TP53 wild type samples to the samples with a nonsynonymous mutation using a two-sided Wilcoxon signed rank test. Only cancers that had at least 5 samples per group were included. In addition, only samples that were diploid at the miR34a locus were used for the analysis to avoid copy number bias.

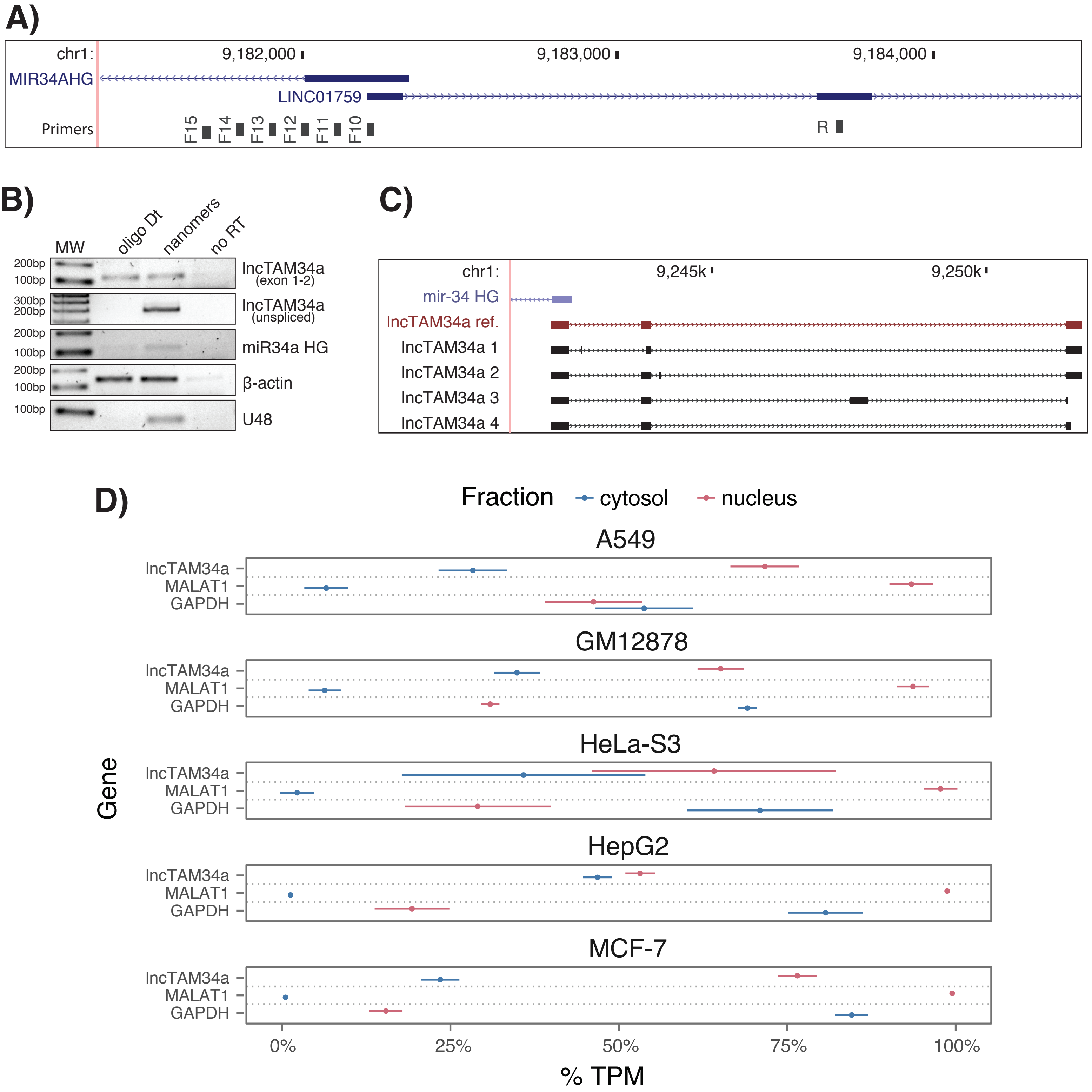

E)

Transcript Name	Coding	Coding Score	Hit number	Hit Score	Frame score	Length	Coverage...	Log Odds Score	Type
lncTAM34a	noncoding (weak)	-1.187350	0	0.0000	0.00000	318	13.43	32.91	full
HOTAIR	noncoding	-0.883468	0	0.0000	0.00000	175	21.89	42.47	full
β-actin	coding	13.662000	250	181.3849	34.26889	1167	62.82	229.54	full

Supplementary Figure 2: A) A schematic representation of the primer placement in the primer walk assay. **B)** Polyadenylation status of spliced and unspliced lncTAM34a in HEK293T cells. **C)** Sequencing results from the analysis of lncTAM34a isoforms in U2OS cells. lncTAM34a ref. refers to the full-length transcript as defined by the 3'-RACE and the primer walk assay. **D)** Analysis of coding potential of the lncTAM34a transcript using the Coding-potential Calculator. **E)** RNAseq data from five fractionated cell lines in the ENCODE project showing the percentage of transcripts per million (TPM) for lncTAM34a. MALAT1 (nuclear localization) and GAPDH (cytoplasmic localization) are included as fractionation controls. Points represent the mean and horizontal lines represent the standard deviation from two biological replicates.

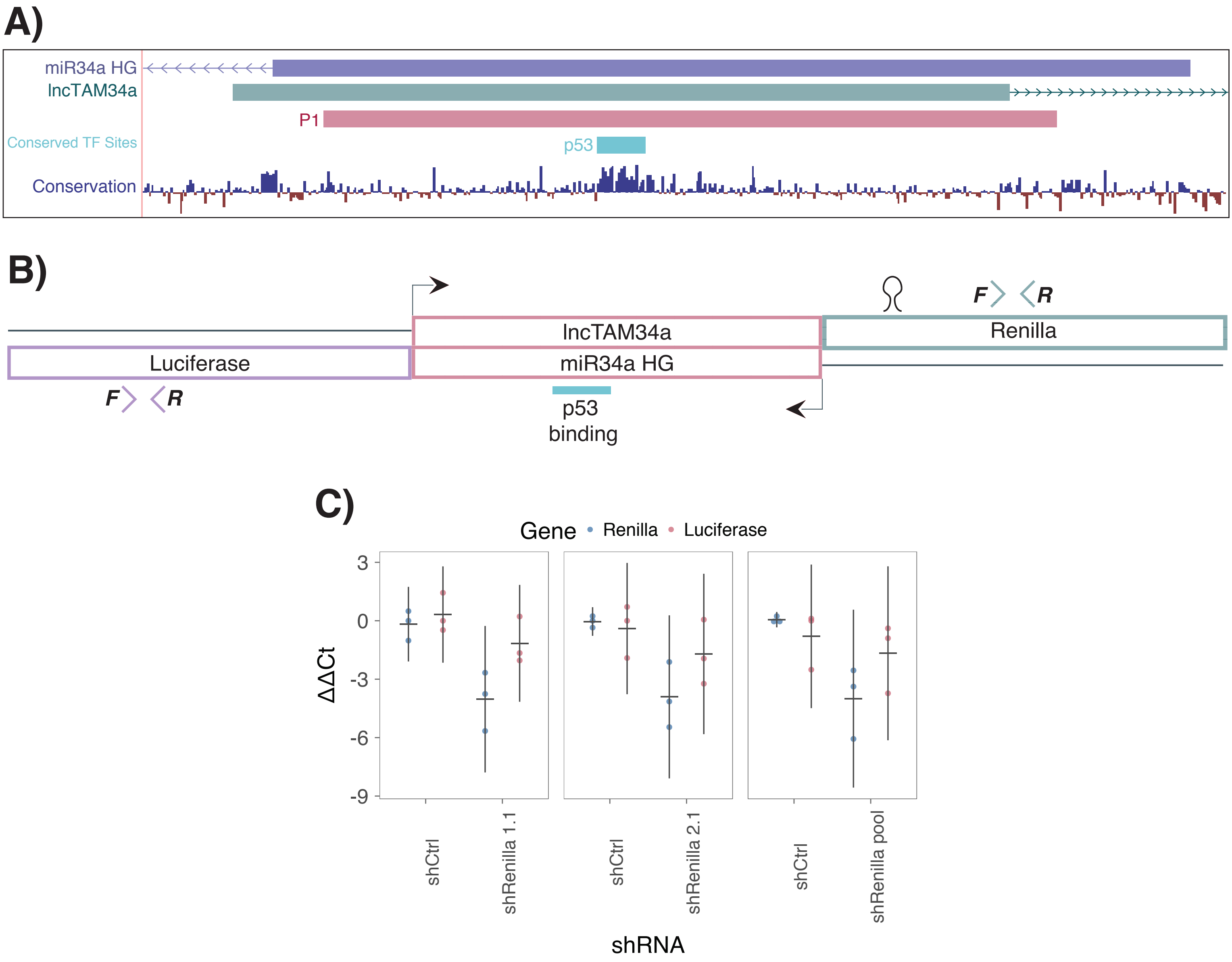

Supplementary Figure 3: A) A UCSC genome browser illustration indicating the location of the promoter region cloned into the p1 construct including the conserved TP53-binding site. **B)** A representative picture of the p1 construct including forward (F) and reverse (R) primer locations and the renilla shRNA targeting site. **C)** HEK293T cells were co-transfected with the p1 construct and either shRenilla or shControl. Renilla and luciferase levels were measured with QPCR 48 hours after transfection. Individual points represent independent experiments, error bars show the 95% CI, and black horizontal lines represent the mean. The experiment was performed in biological triplicate.

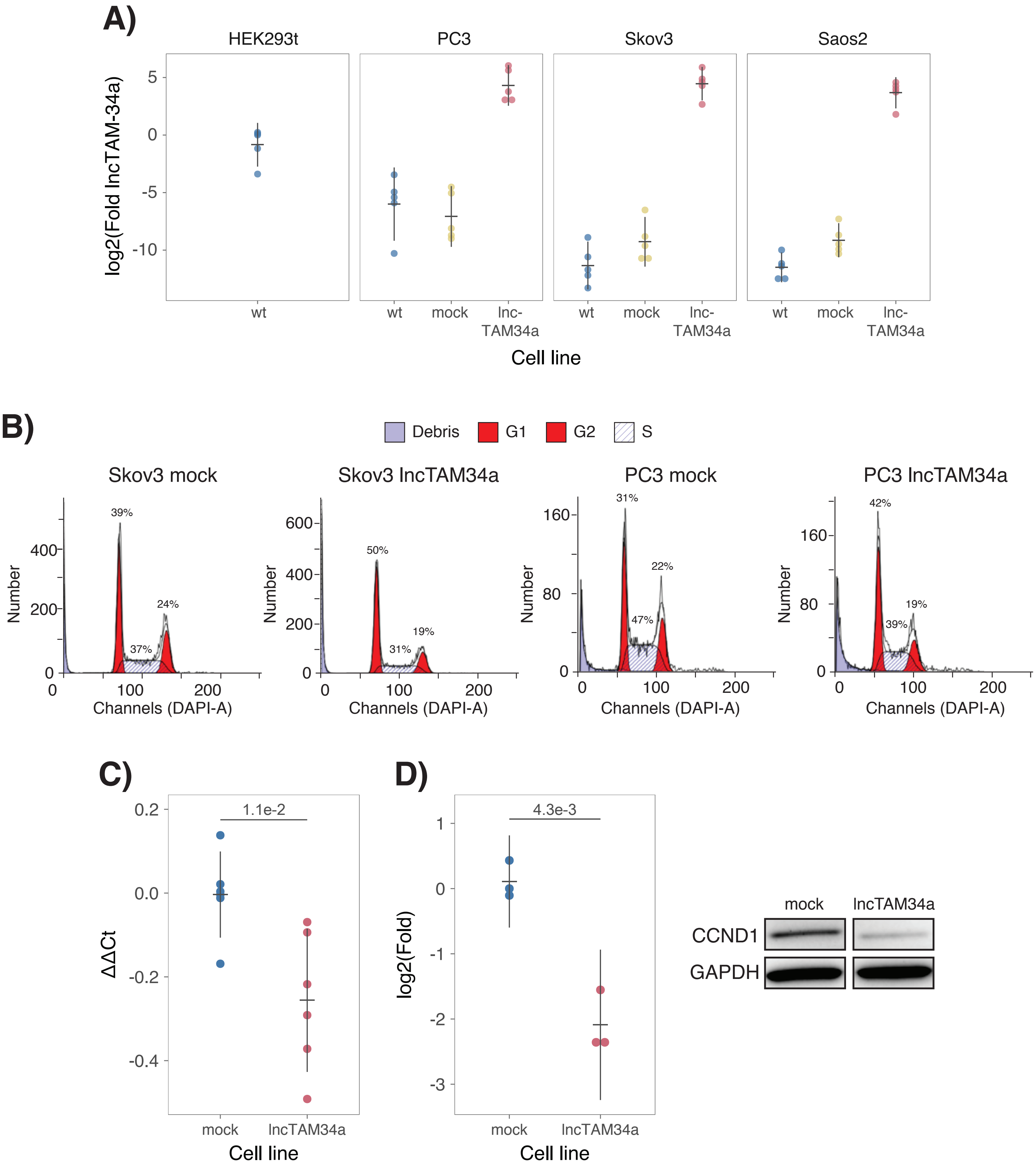

Supplementary Figure 4: A) Comparison of lncTAM34a expression in HEK293T cells (high endogenous lncTAM34a), and the wild-type (wt), mock, and lncTAM34a over-expressing stable cell lines. Individual points represent results from independent experiments, error bars show the 95% CI, and black horizontal lines represent the mean. **B)** Representative flow cytometry analysis from the cell cycle analysis in Figure 3B. CCND1 expression **(C)** and western blot quantification of protein levels **(D)** in lncTAM34a over-expressing PC3 stable cell lines. A representative experiment from the protein quantification experiment is included. Individual points represent results from independent experiments, error bars show the 95% CI, black horizontal lines represent the mean, and P values are shown over long horizontal lines indicating the comparison tested. Experiments were performed in biological sextuplets **(C)** or triplicates **(D)**.

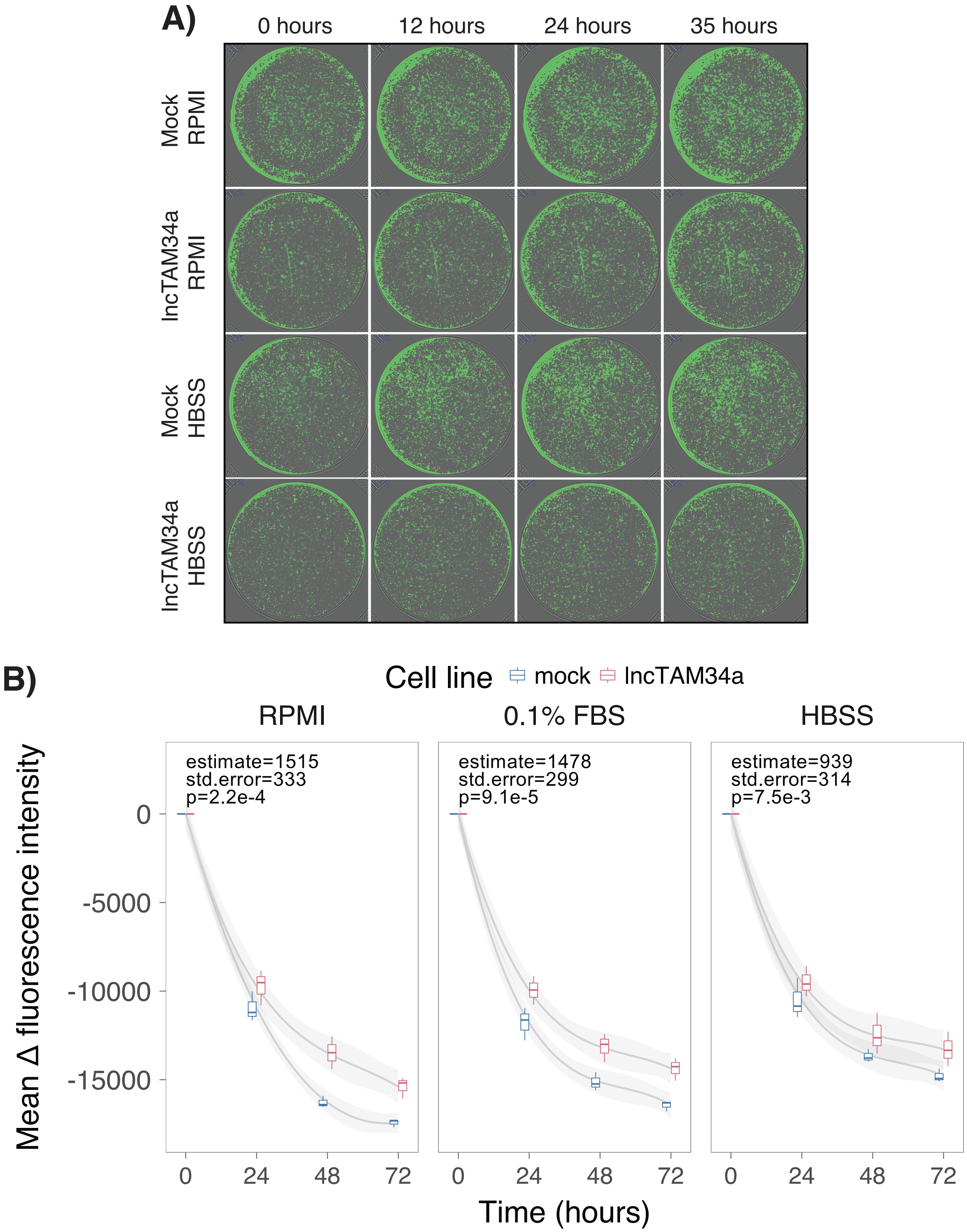

Supplementary Figure 5: A) Representative pictures from the growth analysis shown in Figure 3C. The green color indicates the surface covered by cells as detected by the analysis software. **B)** The effects of miR34a asRNA overexpression on proliferation in normal and starvation conditions in the PC3 prostate cancer cell line. The y-axis illustrates the mean difference (Δ; Time - Time 0) in flouresence intensity for each biological replicate and condition where decreased flouresence intensity indicates increased proliferation. Boxplots show the distribution of the mean differences from each of the biological replicates (n = 3). Grey lines indicate the polynomial regression model with the shadows indicating the 95% confidence intervals. Estimates, standard error (std.error), and P values for the cell line covariate for each model are indicated in the upper left hand corner.

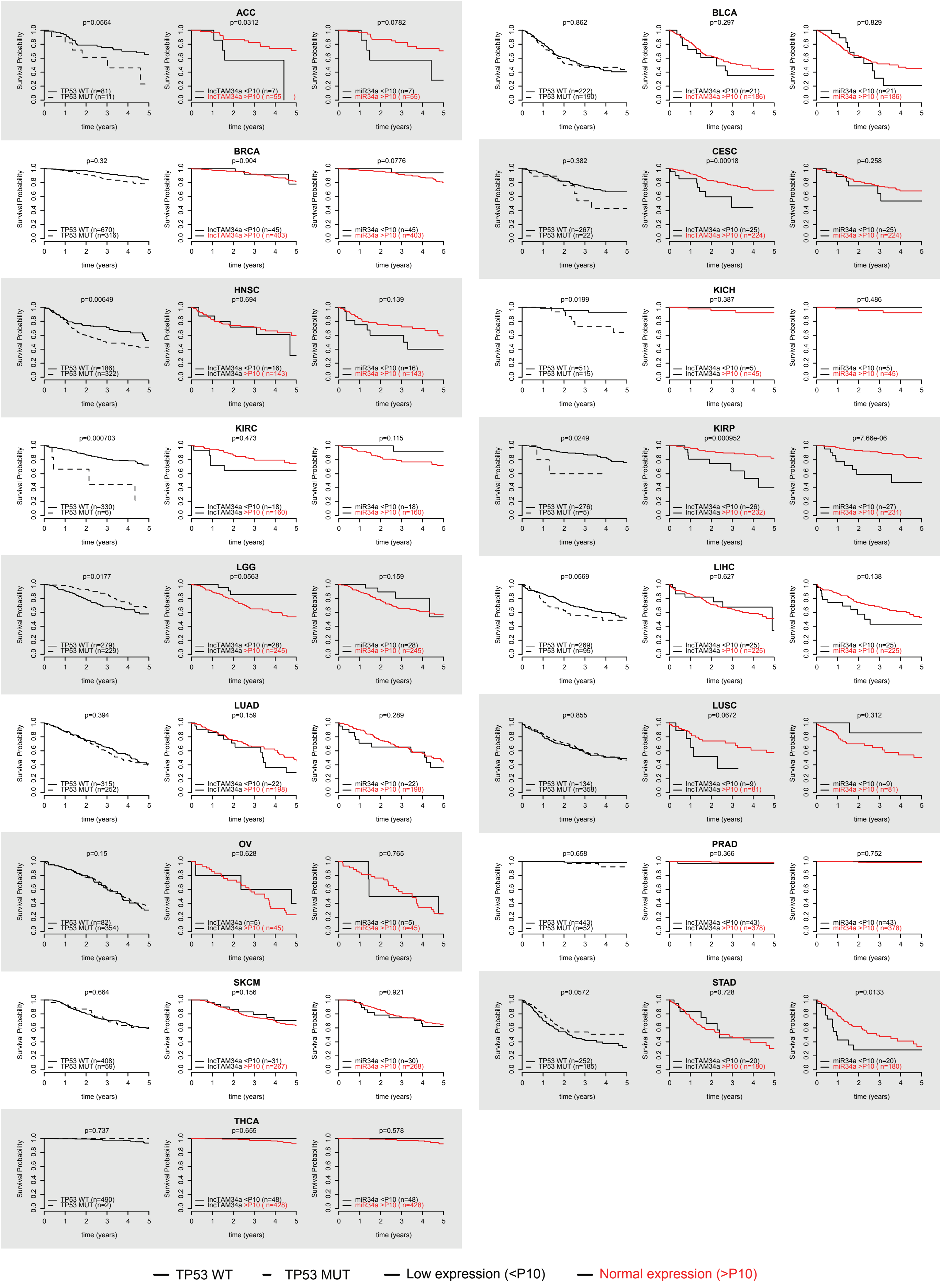

Supplementary Figure 6: Survival analysis in 17 cancers from TCGA. Kaplan-Meier survival curves comparing the survival of TP53-mutated samples (left), low lncTAM34a expression (middle) and low miR34a expression (right) to control samples in 17 cancer types from TCGA. Low expression was defined as TP53 non-mutated samples having expression values in the bottom 10th percentile.

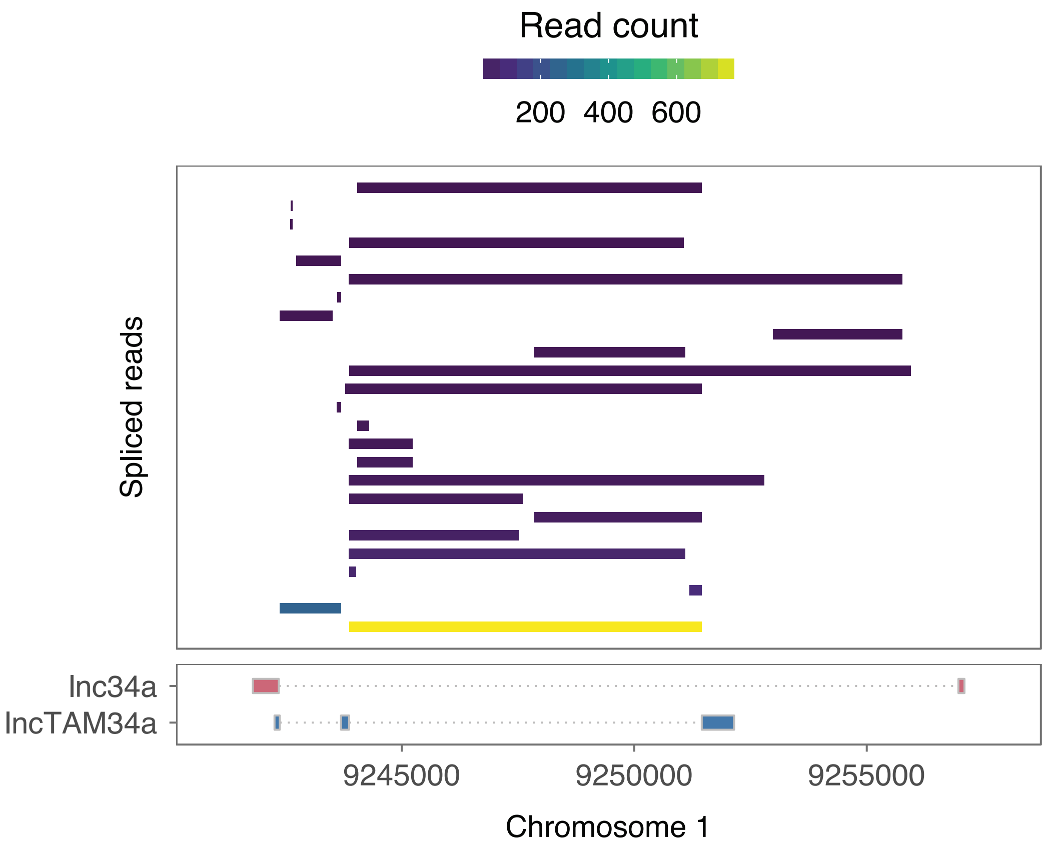

Supplementary Figure 7: All available whole cell (i.e. non-fractionated) spliced read data originating from the Cold Spring Harbor Lab in the ENCODE project for 38 cell lines was downloaded from the UCSC genome browser. Of these cell lines, 36 had spliced reads mapping to the plus strand of chromosome 1 and in the region between the Lnc34a start (9241796) and transcription termination (9257102) site (note that lncTAM34a resides totally within this region). Splice junctions from the following cell lines were included in the final figure: A549, Ag04450, Bj, CD20, CD34 mobilized, Gm12878, H1hesc, Haoaf, Haoec, Hch, Helas3, Hepg2, Hfdpc, Hmec, Hmepc, Hmscat, Hmscbm, Hmscuc, Hob, Hpcpl, Hpiepc, Hsavec, Hsmm, Huvec, Hvmf, Hwp, Imr90, Mcf7, Monocd14, Nhdf, Nhek, Nhemfm2, Nhemm2, Nhlf, Skmc, and Sknsh. All splice junctions were included in the figure and coloured according to the number of reads corresponding to each (top panel). In cases where the exact same read was detected multiple times the read count was summed and represented as one read in the figure. lncTAM34a and Lnc34a transcripts are represented for reference (bottom panel).

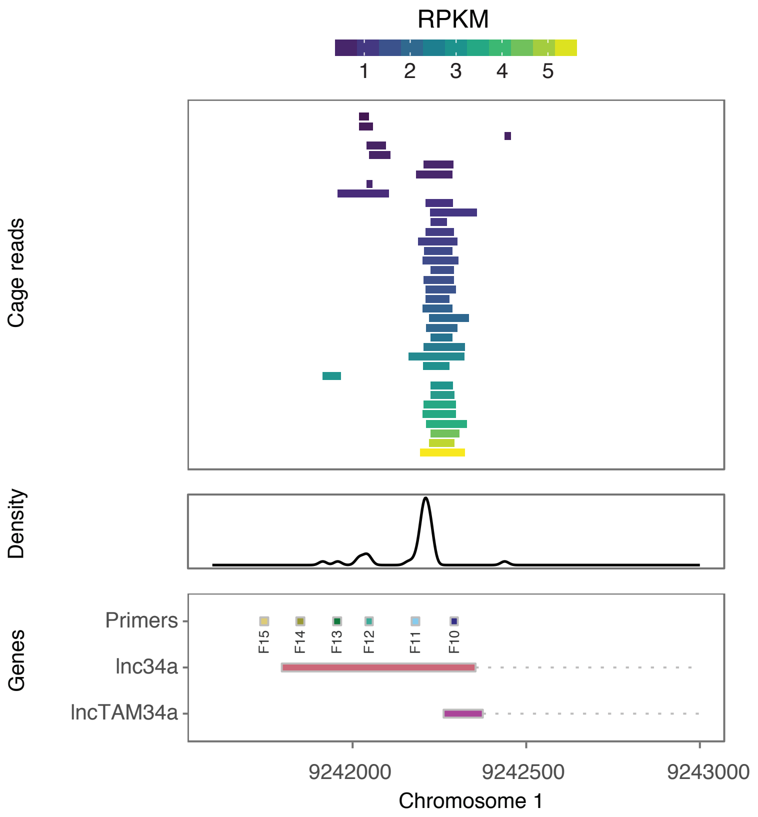

Supplementary Figure 8: All available CAGE data from the ENCODE project for 36 cell lines was downloaded from the UCSC genome browser for genome version hg19. Of these, 28 cell lines had CAGE transcription start sites mapping to the plus strand of chromosome 1 and in regions corresponding to 200 base pairs upstream of the Lnc34a start site (9241796 - 200) and 200 base pairs upstream of the GENCODE annotated lncTAM34a start site (9242263 + 200). These cell lines included: HFDPC, H1-hESC, HMEpC, HAoEC, HPIEpC, HSaVEC, GM12878, hMSC-BM, HUVEC, AG04450, hMSC-UC, IMR90, NHDF, SK-N-SH_RA, BJ, HOB, HPC-PL, HAoAF, NHEK, HVMF, HWP, MCF-7, HepG2, hMSC-AT, NHEM.f_M2, SkMC, NHEM_M2, and HCH. In total 74 samples were included. 17 samples were polyA-, 47 samples were polyA+, and 10 samples were total RNA. In addition, 34 samples were whole cell, 15 enriched for the cytosolic fraction, 10 enriched for the nucleolus, and 15 enriched for the nucleus. All CAGE reads were plotted and the RPKM of the individual reads was used to colour each read to indicate their relative abundance (top panel). In addition, a density plot (middle panel) shows the distribution of the CAGE reads in the specified interval and the transcription start regions for Lnc34a and lncTAM34a as well as primer positions from the primer walk assay (bottom panel).

An unannotated transcript, Lnc34a, arising from the antisense orientation of the miR34a locus and with a transcription start site >250 bp upstream of the annotated lncTAM34a start site, has been previously reported in a study examining colorectal cancer (Wang et al. 2016). Among the findings in Wang et al. the authors discover that Lnc34a negatively regulates miR34a expression via recruitment of DNMT3a, PHB2, and HDAC1 to the miR34a promoter. Although the Lnc34a and lncTAM34a transcripts share some sequence similarity, we believe them to be separate RNAs transcripts. Furthermore, Lnc34a may be highly context dependent and potentially only expressed at biologically significant levels in colon cancer stem cells, or other stem-like cells, in agreement with the conclusions drawn in the paper.

Several lines of evidence point to the fact that lncTAM34a and Lnc34a are not the same transcript and, in addition, that Lnc34a expression may be confined to a highly specific subset of colorectal cancer stem cells (CCSC). First, we were unable to detect transcription upstream of the 5' start site that was defined in the primer walk assay (Fig. 1E and Supplementary Fig. 1B) although the reported Lnc34a start site is >250 base pairs upstream of the F12 primer used in this assay. This could simply be due to the fact that Lnc34a is not expressed in HEK293t cells in which the assay was performed. To further investigate the existence of transcription start sites in the antisense orientation of the miR34a locus, we interrogated CAGE data from 28 cell lines. The results show a high density of CAGE tags aligning to the region corresponding to the annotated lncTAM34a start site (**Supplementary Figure 8**). Several additional peaks, albeit with a much lower average expression, aligns slightly upstream of the annotated lncTAM34a start site, one of which, corresponds to the upstream start site detected in our primer walk analysis (Figure 1e). Despite this, we find no CAGE tags aligning at or upstream of the transcription start site of the Lnc34a transcript. This potentially indicates that Lnc34a is tightly regulated and specifically expressed in the CCSC context, as suggested by the authors. An alternative intrepretation could be that Lnc34a expression is present in a subset of the examined cell lines although at levels too low to be detected. Finally, Lnc34a may not be 5'-capped precluding its detection by CAGE.

In order to detect Lnc34a expression in a manner that is not dependant on 5'-capping, we proceeded to examine spliced RNA sequencing reads from 36 cell lines, taking advantage of the fact that Lnc34a has an exon which is not present in any annotated or PCR cloned lncTAM34a isoforms. These results indicate that, although splice junctions corresponding to the annotated lncTAM34a transcript and multiple isoforms found via PCR cloning were detected, the data give no support for the presence of the splice junction between the first and second exon of Lnc34a (**Supplementary Figure 7**). In summary, these results indicate that Lnc34a is unlikely to represent the same asRNA transcript as lncTAM34a and that its expression may be confined to CCSCs.

In addition, there are several other lines of evidence indicating that the asRNA described in our paper is distinct from Lnc34a. We noted several relevant comments in the public review that was published in conjunction with the work by Wang et al. The authors mention, and provide data, indicating that Lnc34a expression is not changed upon ectopic expression of TP53. In contrast, lncTAM34a is strongly regulated by TP53 as the evidence shows in our, as well as, others findings (Léveillé 2015, Rashi-Elkeles 2014, Hünten 2015, Ashouri 2016, Kim 2017). Furthermore, Wang et al. also mention in the public review that Lnc34a has a low expression level in HCT116 cells although we detect robust expression of lncTAM34a in this cell type (Figure 1b). In summary, these results indicate that Lnc34a expression is not present in the cell types examined where there exists strong evidence for the presence lncTAM34a and, for these reasons, we believe lncTAM34a and Lnc34a to be individual antisense RNA transcripts.
