# Supplementary material for: An antisense RNA capable of modulating the expression of the tumor suppressor microRNA-34a
Source: Cell Death Dis. 2018 Jul 3;9(7):736. doi: 10.1038/s41419-018-0777-5 (PMC6030072; doi:10.1038/s41419-018-0777-5)
Supplement: Supplementary file 2 — Supplementary Document 2 [file 41419_2018_777_MOESM2_ESM.pdf]

Supplementary Document 2

Figure 1b

name	sequence
miR34a asRNA F1	AGC GGC ATC TCC TCC ACC TGA AA
miR34a asRNA R1	TTG CCT CGT GAG TCC AAG GAG AAT
miR34a HG F	TCT GCT CCA GTG GCT GAT GAG AAA
miR34a HG R	GTT CAC TGG CCT CAA AGT TGG CAT
ß-actin Fwd	AGG TCA TCA CCA TTG GCA ATG AG
ß-actin Rev	CTT TGC GGA TGT CCA CGT CA

Figure 1d

name	sequence
miR34a asRNA F10	ACG CGT CTC TCC AGC CCG GGA T
polyT T7 FAM	CAG TGA ATT GTA ATA CGA CTC ACT ATA GGG ACA TCC GTA GCT CGT CCA GGA CCC TTT TTT TTT TTT TTT TTT VN
miR34a asRNA F1	AGC GGC ATC TCC TCC ACC TGA AA
FAM primer	CCG TAG CTC GTC CAG GAC CC

Figure 2a

name	sequence
ß-actin Fwd	AGG TCA TCA CCA TTG GCA ATG AG
ß-actin Rev	CTT TCG GGA TGT CCA CGT CA
miR34a HG F	TCT GCT CCA GTG GCT GAT GAG AAA
miR34a HG R	GTT CAC TGG CCT CAA AGT TGG CAT
miR34a AS F1	AGC GGC ATC TCC TCC ACC TGA AA
miR34a AS R1	TTG CCT CGT GAG TCC AAG GAG AAT

Figure 2b

name	sequence
ß-actin Fwd	AGG TCA TCA CCA TTG GCA ATG AG
ß-actin Rev	CTT TCG GGA TGT CCA CGT CA
miR34a HG F	TCT GCT CCA GTG GCT GAT GAG AAA
miR34a HG R	GTT CAC TGG CCT CAA AGT TGG CAT
miR34a AS F1	AGC GGC ATC TCC TCC ACC TGA AA
miR34a AS R1	TTG CCT CGT GAG TCC AAG GAG AAT

Figure 2c

QPCR primers

name	sequence
ß-actin Fwd	AGG TCA TCA CCA TTG GCA ATG AG
ß-actin Rev	CTT TCG GGA TGT CCA CGT CA
miR34a HG F	TCT GCT CCA GTG GCT GAT GAG AAA
miR34a HG R	GTT CAC TGG CCT CAA AGT TGG CAT
miR34a AS F1	AGC GGC ATC TCC TCC ACC TGA AA
miR34a AS R1	TTG CCT CGT GAG TCC AAG GAG AAT

Figure 2d

name	sequence
Luc setII F	AAG ATT CAA AGT GCG CTG CTG
Luc setII R	TTG CCT GAT ACC TGG CAG ATG
Renilla pBiDir F1	TAA CGC GGC CTC TTC TTA TTT
Renilla pBiDir R1	GAT TTG CCT GAT TTG CCC ATA
ß-actin Fwd	AGG TCA TCA CCA TTG GCA ATG AG
ß-actin Rev	CTT TGC GGA TGT CCA CGT CA

Figure 2e

name	sequence
Luc setII F	AAG ATT CAA AGT GCG CTG CTG
Luc setII R	TTG CCT GAT ACC TGG CAG ATG
Renilla pBiDir F1	TAA CGC GGC CTC TTC TTA TTT
Renilla pBiDir R1	GAT TTG CCT GAT TTG CCC ATA
ß-actin Fwd	AGG TCA TCA CCA TTG GCA ATG AG
ß-actin Rev	CTT TGC GGA TGT CCA CGT CA

Figure 3a

Cloning primers

name	sequence
miR34aAS cloning F4	ACG CGT CTC TCC AGC CCG GGA T
miR34aAS cloning Ex3_1	AAT GAT GGC CGC AAC TAA TGA CGG

QPCR primers

name	sequence
ß-actin Fwd	AGG TCA TCA CCA TTG GCA ATG AG
ß-actin Rev	CTT TCG GGA TGT CCA CGT CA
miR34a AS F1	AGC GGC ATC TCC TCC ACC TGA AA
miR34a AS R1	TTG CCT CGT GAG TCC AAG GAG AAT

Figure 3d

name	sequence
miR34a ChIP F1	AAA GTT TGC AAA GAA GGA GGC GGG
miR34a ChIP R1	AGG GAA GAA AGA ACT AGC CGA GCA

Supplementary Figure 2a

name	sequence
miR34a AS F10	ACG CGT CTC TCC AGC CCG GGA T
miR34a AS F11	ATC TGC GTG GTC ACC GAG AAG CA
miR34a AS F12	CGC ACG GAC TGA GAA ACA CAA G
miR34a AS F13	ACG GAG GCT ACA CAA TTG AAC AGG
miR34a AS F14	AGG GAA GAA AGA ACT AGC CGA GCA
miR34a AS F15	CAT TTG CTG CAA TAT CAC CGT GGC
miR34a AS R1	TTG CCT CGT GAG TCC AAG GAG AAT

Supplementary Figure 2b

name	sequence
miR34a AS F1	AGC GGC ATC TCC TCC ACC TGA AA
miR34a AS R1	TTG CCT CGT GAG TCC AAG GAG AAT
miR34a AS int1 R1	TGC GCA AAC TAC GCG CTC T
miR34a HG F	TCT GCT CCA GTG GCT GAT GAG AAA
miR34a HG R	GTT CAC TGG CCT CAA AGT TGG CAT
ß-actin Fwd	AGG TCA TCA CCA TTG GCA ATG AG
ß-actin Rev	CTT TGC GGA TGT CCA CGT CA
U48 F	AGT GAT GAT GAC CCC AGG TA
U48 R	GGT CAG AGC GCT GCG GTG AT

Supplementary Figure 2c

name	sequence
miR34a AS F12	AAA CAC AAG CGT TTA CCT GGG TGC
miR34a AS R1	TTG CCT CGT GAG TCC AAG GAG AAT
miR34a AS R2	ATA GGT TCA TTT GCC CGA TGT GCC
miR34a AS R3	CCA CAG CTG TTG CTT CTG AAT GCT
miR34a AS Ex3 R1	TGA TGG CCG CAA CTA ATG ACG GAT

Supplementary Figure 3c

name	sequence
Luc setII F	AAG ATT CAA AGT GCG CTG CTG
Luc setII R	TTG CCT GAT ACC TGG CAG ATG
Renilla pBiDir F1	TAA CGC GGC CTC TTC TTA TTT
Renilla pBiDir R1	GAT TTG CCT GAT TTG CCC ATA
ß-actin Fwd	AGG TCA TCA CCA TTG GCA ATG AG
ß-actin Rev	CTT TGC GGA TGT CCA CGT CA

Supplementary Figure 4a

Cloning primers

name	sequence
miR34aAS cloning F4	ACG CGT CTC TCC AGC CCG GGA T
miR34aAS cloning Ex3_1	AAT GAT GGC CGC AAC TAA TGA CGG

QPCR primers

name	sequence
ß-actin Fwd	AGG TCA TCA CCA TTG GCA ATG AG
ß-actin Rev	CTT TCG GGA TGT CCA CGT CA
miR34a AS F1	AGC GGC ATC TCC TCC ACC TGA AA
miR34a AS R1	TTG CCT CGT GAG TCC AAG GAG AAT

Supplementary Figure 4c

name	sequence
CCND1 Fwd	CGT GGC CTC TAA GAT GAA GG
CCND1 Rev	CTG GCA TTT TGG AGA GGA AG
ß-actin Fwd	AGG TCA TCA CCA TTG GCA ATG AG
ß-actin Rev	CTT TGC GGA TGT CCA CGT CA
